# Patterns of antimicrobial use among hospitalized Veterans with and without a penicillin-class allergy

**DOI:** 10.1017/ash.2025.11

**Published:** 2025-02-25

**Authors:** Funnce Liu, Hang Hoang-Nguyen, Erin Ham, McKenna C. Eastment, Luis G. Tulloch-Palomino

**Affiliations:** 1 Pharmacy Services, VA Puget Sound Health Care System, Seattle, WA, USA; 2 General Medical Services, VA Puget Sound Health Care System, Seattle, WA, USA; 3 Division of General Internal Medicine, Department of Medicine, University of Washington School of Medicine, Seattle, WA, USA; 4 Hospital and Specialty Medicine, VA Puget Sound Health Care System, Seattle, WA, USA; 5 Division of Allergy and Infectious Diseases, Department of Medicine, University of Washington School of Medicine, Seattle, WA, USA

## Abstract

**Objective:**

To explore the progress that the Veterans Health Administration has made to minimize the impact of the penicillin (PCN) allergy label, we determined the proportion of Veterans who reported a PCN-class allergy at the time of hospitalization and described antibiotic use in hospitalizations with and without a PCN-class allergy.

**Design:**

Cross-sectional study.

**Participants:**

National sample of 6,541,299 acute care admissions between 2011 and 2022.

**Methods:**

We calculated the prevalence of PCN-class allergies on admission and used Poisson regression to compare patterns of antibiotic use between hospitalizations with and without a PCN-class allergy.

**Results:**

The prevalence of PCN-class allergies on admission decreased from 12.99% to 11.20%. Use of cefazolin and non-pseudomonal third-generation cephalosporins increased regardless of PCN-class allergy status (“PCN-class allergy only” +11.46%, “No antibiotic allergy” +4.92%). The prevalence ratio (PR) for antibiotic use in hospitalizations with a PCN-class allergy compared to hospitalizations without antibiotic allergies, decreased for anti-Methicillin Resistant *Staphylococcus aureus* agents (1.26 [1.25, 1.28] to 1.15 [1.13, 1.17]), carbapenems (1.59 [1.54, 1.65] to 1.47 [1.41, 1.53]), and aztreonam (23.89 [22.45, 25.43] to 17.57 [15.90, 19.42]); and increased for fluoroquinolones (1.58 [1.56, 1.60] to 2.15 [2.09, 2.20]).

**Conclusions:**

Prevalence of PCN-class allergies is declining and narrow-spectrum βL use is rising among hospitalized Veterans. Prescribing differences are decreasing between hospitalizations with and without a reported PCN-class allergy, except for aminoglycosides, clindamycin, and fluoroquinolones. These findings can serve to identify areas of focus for future analyses or interventions related to the impact of the PCN allergy label on antibiotic selection.

## Introduction

Between 2000 and 2014, 13% of hospitalized Veterans had a reported allergy to a natural or synthetic penicillin (PCN).^1^ After allergy testing, nearly 90% of individuals with a reported PCN allergy are found to tolerate PCN and other β-lactams (βLs).^2,3^ The PCN allergy label is associated with increased broad-spectrum antibiotic use,^4–6^ delays in antibiotic administration,^7^ increased acute kidney injury, and *Clostridioides difficile* infection,^8–10^ increased colonization with multi-drug resistant organisms,^8^ suboptimal clinical outcomes (eg, surgical site infection, readmission, length of stay (LOS), mortality),^4,8–11^ and increased cost.^12,13^ Interventions aimed at evaluating and removing inaccurate PCN allergy labels have been shown to decrease broad-spectrum antibiotic use and cost^14,15^ and are encouraged by society guidelines.^16,17^

Over the past decade, dozens of Veterans Health Administration (VHA) medical centers have implemented PCN allergy evaluation programs.^3,18–27^ However, the aggregate impact of these efforts is unclear because there are no recent estimates of the prevalence of PCN or other βL allergies among hospitalized Veterans and because descriptions of the impact of the PCN allergy label on inpatient antibiotic use at VHA facilities are limited to small single-center studies.^7,28^ To explore the progress that the VHA has made to mitigate the effects of the PCN allergy label, we analyzed a national sample of acute care admissions between 2011 and 2022 to determine the proportion of patients who had a reported PCN-class allergy at the time of admission and compare patterns of inpatient antibiotic use between hospitalizations with and without a reported PCN-class allergy.

## Methods

### Data sources and study population

The VHA is one of the largest integrated healthcare systems in the United States (US), offering inpatient services at 172 facilities across 49 states, the District of Columbia, and Puerto Rico.^29^ The VHA adopted the Veterans Health Information Systems and Technology Architecture (VISTA), a national electronic health record (EHR), in the early 1990s.

Each VHA health system (or station) uses a localized instance of VISTA and data captured in these localized instances is aggregated and stored in standardized data repositories.^30^ For this cross-sectional study, we analyzed allergy, admission, discharge, and bar-code medication administration (BCMA) records abstracted from a relational database created by the Veterans Affairs Informatics and Computing Infrastructure (VINCI) service. We included all acute care admissions between January 1, 2011 and December 31, 2022. We excluded admissions to four VHA facilities that transitioned to the Cerner-Oracle EHR, admissions to non-acute care services (Blind Rehabilitation, Community Living Center (CLC), Domiciliary, Psychiatry, and Respite Care), and admissions with incomplete allergy, admission, or discharge records (Table S1).

### Antibiotic allergy definitions

Because allergy records are not automatically shared between VHA stations, an individual was considered to have an active antibiotic allergy if the allergy label had been entered and remained present in the admitting station’s EHR at the time of admission. If the allergy label had been entered in another station’s EHR but not in the admitting station’s EHR or if the allergy label had been entered in the admitting station’s EHR but discontinued prior to admission, then it was not counted. If the allergy label was present at the time of admission but later discontinued during the same admission, it was counted for the current hospitalization but not for any subsequent hospitalizations (Table S2). Antibiotic allergies were identified by their generic or US brand names and grouped into 25 categories based on pharmacologic class (Table S3). For the comparison of inpatient antibiotic use between hospitalizations with and without a reported PCN-class allergy, the “PCN-class allergy only” group included admissions where the patient reported an allergy to “Penicillin,” “Amoxicillin/Ampicillin,” or “Other Synthetic Penicillin” as their only antibiotic allergy, and the “No antibiotic allergy” group included admissions where the patient did not report any antibiotic allergies.

### Administered antibiotic definitions

Administered antibiotics were identified by their generic or US brand names and categorized into 11 groups based on their pharmacologic class and National Healthcare Safety Network grouping;^31^ 4 non-βL groups (“Aminoglycoside,” “Anti-Methicillin Resistant *Staphylococcus aureus* (MRSA),” “Clindamycin,” and “Fluoroquinolone,”) and 7 βL groups (“Anti-Pseudomonal (Anti-Ps) Cephalosporin,” “Anti-Ps Penicillin,” “Carbapenem,” “Monobactam,” “Narrow-spectrum (NS) Structurally Different Cephalosporin,” “NS Structurally Similar Cephalosporin,” and “NS Penicillin”) (Table S4). The “Anti-MRSA” group did not include ceftaroline. NS cephalosporins were considered structurally different if they did not share side-chain groups with PCN or Amoxicillin/Ampicillin.^32^ If a patient received a dose of any antibiotic in a group on a calendar day, they were considered to have received a day of that antibiotic group.

### Statistical analysis

We analyzed each admission as a separate observation. Therefore, patients with multiple admissions contributed multiple observations, each unique in regard to reported allergy history. To compare inpatient antibiotic use between hospitalizations in the “PCN-class allergy only” and “No antibiotic allergy” groups, we used Poisson regression with robust standard errors to obtain the prevalence ratio (PR) for antibiotic use across six time periods (2011-2012, 2013-2014, 2015-2016, 2017-2018, 2019-2020, and 2021-2022). Analyses were conducted in Stata version 18.1 (College Station, TX). All tests were two-sided, and *P* < 0.05 was considered statistically significant.

### Ethical statement

The study was approved by the institutional review board of the Veterans Affairs (VA) Puget Sound Health Care System. The requirement for informed consent was waived.

## Results

We included 6,541,299 acute care admissions. The average admission age was 67.26 years (± 13.00), and the birth sex was male in 6,193,628 (94.68%) admissions. The most frequent admitting service was Medicine (4,877,663; 74.57%), and the average LOS was 5.31 days (± 16.22) (Table 1).

**Table 1.** Percent of acute care admissions between 2011 and 2022 where patients reported a β-lactam allergy as their only antibiotic allergy or as one of multiple antibiotic allergies

	All Admissions
	N = 6,541,299
Patients admitted	2,208,079
Mean LOS (days) ± SD	5.31 ± 16.22
Age at admission (years) ± SD	67.26 ± 13.00
Male sex at birth	6,193,628 (94.68%)
**Region^1^**	
1	1,470,837 (22.49%)
2	1,448,009 (22.14%)
3	1,429,072 (21.85%)
4	1,064,920 (16.28%)
5	1,128,461 (17.25%)
**Admitting service**	
Intermediate Medicine	19,597 (0.30%)
Medicine	4,877,663 (74.57%)
Neurology	43,917 (0.67%)
Rehabilitation Medicine	31,736 (0.49%)
Spinal Cord Injury	74,667 (1.14%)
Surgery	1,493,719 (22.84%)
**Single allergy reported at admission**	
Any penicillin-class allergy	587,076 (8.97%)
Penicillin	516,665 (7.90%)
Amoxicillin/Ampicillin^2^	56,506 (0.86%)
Other Synthetic Penicillin^2^	13,905 (0.21%)
Any cephalosporin allergy	47,123 (0.72%)
Cefazolin	4,576 (0.07%)
Cephalosporin, 1^st^ or 2^nd^ generation	31,920 (0.49%)
Cephalosporin, 3^rd^ generation^2^	7,787 (0.12%)
Cephalosporin, 4^th^ generation	2,434 (0.04%)
Cephalosporin, other or unspecified	406 (0.01%)
Monobactam	106 (<0.01%)
Carbapenem^2^	2,092 (0.03%)
Unspecified β-lactam	1,115 (0.02%)
**Multiple allergies reported at admission**^3^	
Any penicillin-class allergy	216,338 (3.31%)
Penicillin	176,681 (2.70%)
Amoxicillin/Ampicillin^2^	44,273 (0.68%)
Other Synthetic Penicillin^2^	13,537 (0.21%)
Any cephalosporin allergy	54,600 (0.83%)
Cefazolin	5,472 (0.08%)
Cephalosporin, 1^st^ or 2^nd^ generation	36,145 (0.55%)
Cephalosporin, 3^rd^ generation^2^	10,787 (0.16%)
Cephalosporin, 4^th^ generation	3,773 (0.06%)
Cephalosporin, other or unspecified	947 (0.01%)
Monobactam	928 (0.01%)
Carbapenem^2^	3,460 (0.05%)
Unspecified β-lactam	1356 (0.02%)

Abbreviations: LOS, Length of stay; SD, Standard deviation.

1 Region 1: CT, DC, DE, MA, MD, ME, NC, NH, NJ, NY, PA, RI, VA, VT, WV; Region 2: AL, FL, GA, KY, PR, SC TN; Region 3: IA, IL, IN, KS, KY, MI, MN, MO, ND, NE, OH, SD, WI; Region 4: AR, CO, LA, MS, MT, OK, TX, UT, WY; Region 5: AZ, CA, HI, ID, NM, NV, OR, WA.

2 ± β-lactamase inhibitor (BLI).

3 Includes patients that reported multiple antibiotic allergies at the time of admission; could report a single or multiple β-lactam allergies as part of those multiple antibiotic allergies.

**Figure 1.**
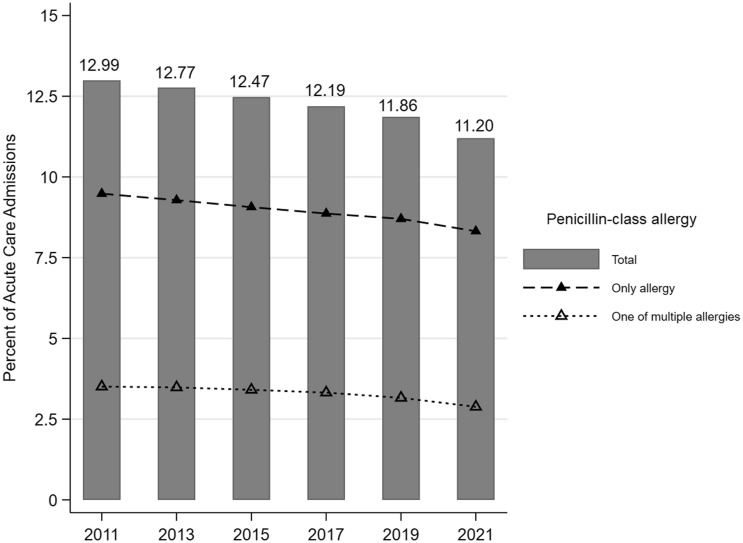
Percent of acute care admissions where a penicillin-class allergy was reported either as the only antibiotic allergy or as one of multiple antibiotic allergies. * Each bar represents a 2-year period. The x-axis shows the first year of that 2-year period (eg, 2011 includes 2011 and 2012).

An allergy to a PCN-class agent was reported in 803,414 (12.28%) admissions either as the only antibiotic allergy (587,076; 8.97%) or as one of multiple antibiotic allergies (216,338; 3.31%) (Table 1). The prevalence of reported PCN-class allergies decreased from 12.99% in 2011-2012 to 11.20% in 2021-2022 (Figure 1). The same trend was observed for admission age ≥65 years and across all regions; however, the prevalence of reported PCN-class allergies remained relatively stable for admission age <65 years (Figure S1-S2). For admissions where a single allergy was reported, an allergy to a cephalosporin-, monobactam-, and carbapenem-class agent was reported in 47,123 (0.72%), 106 (<0.01%), and 2,092 (0.03%) admissions, respectively (Table 1). For admissions where multiple allergies were reported, an allergy to a cephalosporin was reported in 54,600 (0.83%), an allergy to a monobactam was reported in 928 (0.01%), and an allergy to a carbapenem was reported in 3,460 (0.05%) (Table 1). Prevalence of non-β-lactam antibiotic allergies are shown in Table S5.

To compare inpatient antibiotic use between hospitalizations with and without a reported PCN-class allergy, we identified 587,076 admissions in the “PCN-class allergy only” group and 5,246,497 admissions in the “No antibiotic allergy” group (Table 2). Compared to hospitalizations in the “No antibiotic allergy” group, “PCN-class allergy only” admissions had higher frequency of Aminoglycoside (1.24 vs 0.88%; PR 1.40 [95% CI 1.37, 1.43]), Anti-MRSA (17.76 vs 14.45%; PR 1.23 [95% CI 1.22, 1.24]), Clindamycin (6.60 vs 1.57%; PR 4.21 [95% CI 4.16, 4.26]), Fluoroquinolone (17.22 vs 9.93%; PR 1.73 [95% CI 1.72, 1.75]), Anti-Ps Cephalosporin (5.91 vs 4.12%, PR 1.44 [95% CI 1.42, 1.45]), Carbapenem (3.61 vs 2.17%; PR 1.67 [95% CI 1.64, 1.69]), and Monobactam (2.72 vs 0.12%; PR 22.76 [95% CI 22.11, 23.44]) use; and lower frequency of Anti-Ps Penicillin (2.67 vs 12.06%; PR 0.22 [95% CI 0.22, 0.23]), NS Different Cephalosporin (12.96 vs 21.93%; PR 0.59 [95% CI 0.59, 0.60]), NS Similar Cephalosporin (1.01 vs 1.54%; PR 0.65 [95% CI 0.64, 0.67]), and NS Penicillin (1.39 vs 6.29%; PR 0.22 [95% CI 0.22, 0.23]) use (Table 2).

**Table 2.** Total prevalence and prevalence ratio of antibiotic use in acute care admissions between 2011 and 2022 where patients reported a penicillin-class allergy only or no antibiotic allergy at the time of admission

	PCN-class allergy only^1^	No antibiotic allergy	p	PR	95% CI
	N = 587,076	N = 5,246,497			
Mean LOS (± SD)	5.19 (±14.86)	5.31 (±15.89)	<0.001	–	–
Age at admission (± SD)	68.31 (±12.74)	66.96 (±13.03)	<0.001	–	–
Male sex at birth	551,333 (93.91%)	5,022,456 (95.73%)	<0.001	–	–
**Region**					
1	131,652 (22.43%)	1,184,831 (22.58%)	<0.001	–	–
2	126,770 (21.59%)	1,165,427 (22.21%)			
3	132,545 (22.58%)	1,134,956 (21.63%)			
4	98,370 (16.76%)	857,725 (16.35%)			
5	97,739 (16.65%)	903,558 (17.22%)			
**Admitting service**					
Intermediate Medicine	1,704 (0.29%)	15,835 (0.30%)	<0.001	–	–
Medicine	445,137 (75.82%)	3,891,348 (74.17%)			
Neurology	3,904 (0.66%)	36,102 (0.96%)			
Rehabilitation Medicine	2,342 (0.40%)	26,766 (0.51%)			
Spinal Cord Injury	5,835 (0.99%)	53,950 (1.03%)			
Surgery	128,154 (21.83%)	1,222,496 (23.30%)			
**Antibiotic group administered**					
Aminoglycoside					
Received n (%)	7,264 (1.24%)	46,365 (0.88%)	<0.001	1.4	1.37, 1.43
Mean Days (± SD)	2.53 (±4.42)	2.54 (±4.38)	0.86	–	–
Anti-MRSA					
Received n (%)	104,260 (17.76%)	758,261(14.45%)	<0.001	1.23	1.22, 1.24
Mean Days (± SD)	4.11 (±5.35)	4.28 (±5.33)	<0.001	–	–
Clindamycin					
Received n (%)	38,762 (6.60%)	82,343 (1.57%)	<0.001	4.21	4.16, 4.26
Mean Days (± SD)	2.85 (±2.62)	3.22 (±2.94)	<0.001	–	–
Fluoroquinolone					
Received n (%)	101,118 (17.22%)	520,971 (9.93%)	<0.001	1.73	1.72, 1.75
Mean Days (± SD)	3.61 (±3.55)	3.51 (±4.64)	<0.001	–	–
Anti-Ps Cephalosporin					
Received n (%)	34,710 (5.91%)	216,153 (4.12%)	<0.001	1.44	1.42, 1.45
Mean Days (± SD)	4.66 (±4.79)	4.50 (±4.64)	<0.001	–	–
Anti-Ps Penicillin					
Received n (%)	15,704 (2.67%)	632,897 (12.06%)	<0.001	0.22	0.22, 0.23
Mean Days (± SD)	4.25 (±4.24)	4.67 (±4.75)	<0.001	–	–
Carbapenem					
Received n (%)	21,191 (3.61%)	113,699 (2.17%)	<0.001	1.67	1.64, 1.69
Mean Days (± SD)	5.61 (±6.75)	5.93 (±7.53)	<0.001	–	–
Monobactam					
Received n (%)	15,984 (2.72%)	6,275 (0.12%)	<0.001	22.76	22.11, 23.44
Mean Days (± SD)	4.31 (±4.05)	3.84 (±4.88)	<0.001	–	–
NS Different Cephalosporin					
Received n (%)	76,088 (12.96%)	1,150,376 (21.93%)	<0.001	0.59	0.59, 0.60
Mean Days (± SD)	3.42 (±3.47)	2.88 (±2.84)	<0.001	–	–
NS Similar Cephalosporin					
Received n (%)	5,913 (1.01%)	80,735 (1.54%)	<0.001	0.65	0.64, 0.67
Mean Days (± SD)	3.25 (±4.76)	3.14 (±4.65)	0.08	–	–
NS Penicillin					
Received n (%)	8,179 (1.39%)	330,182 (6.29%)	<0.001	0.22	0.22, 0.23
Mean Days (± SD)	4.07 (±5.83)	3.99 (±5.59)	0.25	–	–

Abbreviations: SD, Standard deviation; PR, Prevalence Ratio; CI, Confidence Interval; LOS, Length of Stay; MRSA, Methicillin Resistant *Staphylococcus aureus*; Anti-Ps, Antipseudomonal; NS, Narrow-spectrum.

1 Includes acute care admissions where patients reported an allergy to Penicillin, Amoxicillin/Ampicillin (± β-lactamase inhibitor), or other synthetic penicillin-class antibiotics as their only antibiotic allergy at the time of admission.

In “PCN-class allergy only” admissions, use of Aminoglycoside, Anti-MRSA, Clindamycin, Fluoroquinolone, Carbapenem, and Monobactam agents decreased; 1.77, 19.90, 7.87, 24.27, 3.81, and 3.45% in 2011-2012, compared to 0.69, 15.71, 4.17, 8.76, 3.18, and 1.33% in 2021-2022. In contrast, use of Anti-Ps Cephalosporin, Anti-Ps Penicillin, NS Different Cephalosporin, NS Similar Cephalosporin, and NS Penicillin agents increased; 4.12, 2.55, 9.01, 0.75, and 0.93% in 2011-2012, compared to 9.32, 2.81, 20.47, 1.34, and 2.25% in 2021-2022 (Table 3).

**Table 3.** Biannual prevalence and prevalence ratio of antibiotic use in acute care admissions between 2011 and 2022 where patients reported a penicillin-class allergy only or no antibiotic allergy at the time of admission

Antibiotic group administered	2011-2012^1^		2013-2014^1^		2015-2016^1^		2017-2018^1^		2019-2020^1^		2021-2022^1^	
	PCN^2^ N = 108,427	No allergy N = 917,719	PCN^2^ N = 105,747	No allergy N = 912,470	PCN^2^ N = 102,986	No allergy N = 909,198	PCN^2^ N = 100,162	No allergy N = 903,311	PCN^2^ N = 89,333	No allergy N = 822,950	PCN^2^ N = 80,421	No allergy N = 780,849
Aminoglycosides	1.77	1.27	1.47	1.06	1.31	0.94	1.10	0.82	0.88	0.60	0.69	0.53
	1.40 (1.33, 1.47)		1.38 (1.31, 1.46)		1.39 (1.31, 1.47)		1.35 (1.26, 1.43)		1.45 (1.35, 1.56)		1.31 (1.20, 1.43)	
Anti-MRSA	19.90	15.77	19.28	15.31	18.1	14.44	16.65	13.76	16.03	13.57	15.72	13.64
	1.26 (1.25, 1.28)		1.26 (1.24, 1.28)		1.25 (1.24, 1.27)		1.21 (1.19, 1.23)		1.18 (1.16, 1.20)		1.15 (1.13, 1.17)	
Clindamycin	7.87	1.96	7.62	1.90	7.24	1.81	6.73	1.41	5.17	1.16	4.17	1.07
	4.01 (3.91, 4.12)		4.02 (3.91, 4.12)		4.00 (3.89, 4.10)		4.79 (4.65, 4.93)		4.46 (4.31, 4.61)		3.91 (3.76, 4.07)	
Fluoroquinolone	24.27	15.33	22.07	13.43	18.62	11.51	15.54	8.48	10.84	5.43	8.76	4.08
	1.58 (1.56, 1.60)		1.64 (1.62,1.66)		1.61 (1.60, 1.64)		1.83 (1.80, 1.86)		2.00 (1.95, 2.04)		2.15 (2.09, 2.20)	
Anti-Ps Cephalosporin	4.12	2.78	4.29	2.60	5.27	3.75	5.78	3.62	7.85	5.59	9.32	6.94
	1.48 (1.44, 1.53)		1.65 (1.60, 1.70)		1.40 (1.37, 1.44)		1.60 (1.55, 1.64)		1.40 (1.37, 1.44)		1.34 (1.31, 1.37)	
Anti-Ps Penicillin	2.55	13.43	2.64	13.31	2.49	11.42	2.80	12.10	2.83	11.10	2.81	10.72
	0.19 (0.18, 0.20)		0.20 (0.19, 0.21)		0.22 (0.21, 0.23)		0.23 (0.22, 0.24)		0.26 (0.25, 0.27)		0.26 (0.25, 0.27)	
Carbapenem	3.81	2.39	3.89	2.25	3.66	2.22	3.52	1.91	3.46	2.06	3.18	2.17
	1.59 (1.54, 1.65)		1.73 (1.67, 1.79)		1.65 (1.60, 1.71)		1.84 (1.78, 1.91)		1.68 (1.62, 1.75)		1.47 (1.41, 1.53)	
Monobactam	3.45	0.14	3.64	0.14	3.02	0.17	2.54	0.10	1.87	0.08	1.33	0.08
	23.89 (22.45, 25.43)		25.83 (24.25, 27.50)		17.81 (16.76, 18.92)		25.24 (23.41, 27.22)		24.76 (22.59, 27.14)		17.57 (15.90, 19.42)	
NS Different Cephalosporin	9.01	20.27	9.58	20.05	10.73	20.61	13.20	22.02	17.30	24.11	20.47	25.19
	0.44 (0.44, 0.45)		0.48 (0.47, 0.49)		0.52 (0.51, 0.53)		0.60 (0.59, 0.61)		0.72 (0.71, 0.73)		0.81 (0.80, 0.82)	
NS Similar Cephalosporin	0.75	1.41	0.79	1.46	0.92	1.53	1.10	1.60	1.26	1.64	1.34	1.61
	0.53 (0.49, 0.57)		0.54 (0.51, 0.58)		0.60 (0.56, 0.64)		0.69 (0.65, 0.73)		0.77 (0.73, 0.82)		0.84 (0.79, 0.89)	
NS Penicillin	0.93	6.12	1.03	5.92	1.21	5.88	1.43	6.33	1.78	6.69	2.25	6.95
	0.15 (0.14, 0.16)		0.17 (0.16, 0.18)		0.21 (0.20, 0.22)		0.23 (0.22, 0.24)		0.27 (0.25, 0.28)		0.32 (0.31, 0.34)	

Abbreviations: PR, Prevalence Ratio; CI, Confidence Interval; PCN, Penicillin; MRSA, Methicillin Resistant *Staphylococcus aureus*.

1 For “Penicillin-class allergy only” and “No antibiotic allergy” groups, upper rows show percent of admissions where ≥1 dose of an antibiotic from the specified antibiotic group was administered, and lower row shows PR for biannual period.

2 “Penicillin-class allergy only” group: Includes acute care admissions where patients reported an allergy to Penicillin, Amoxicillin/Ampicillin (± β-lactamase inhibitor), or other synthetic penicillin-class antibiotics as their only antibiotic allergy at the time of admission.

In “No antibiotic allergy” admissions, use of Aminoglycoside, Anti-MRSA, Clindamycin, Fluoroquinolone, Anti-Ps Penicillin, Carbapenem, and Monobactam agents decreased; 1.27, 15.77, 1.96, 15.33, 13.43, 2.39, and 0.14% in 2011-2012, compared to 0.53, 13.64, 1.07, 4.08, 10.72, 2.17, and 0.08% in 2021-2022. In contrast, use of Anti-Ps Cephalosporin, NS Different Cephalosporin, NS Similar Cephalosporin, and NS Penicillin agents increased; 2.78, 20.27, 1.41, and 6.12% in 2011-2012, compared to 6.94, 25.19, 1.61, and 6.95% in 2021-2022 (Table 3).

Comparing the 2011-2012 and 2021-2022 time periods, the PR for antibiotic use among “PCN-class allergy only” and “No antibiotic allergy” admissions decreased for the following groups: Anti-MRSA (1.26 [95% CI 1.25, 1.28] to 1.15 [95% CI 1.13, 1.17]), Anti-Ps Cephalosporin (1.48 [95% CI 1.44, 1.53] to 1.34 [95% CI 1.31, 1.37]), Carbapenem (1.59 [95% CI 1.54, 1.65] to 1.47 [95% CI 1.41, 1.53]), and Monobactam (23.89 [95% CI 22.45, 25.43] to 17.57 [95% CI 15.90, 19.42]) (Table 3, Figure S3-S4). The PR increased for the following groups: Fluoroquinolone (1.58 [95% CI 1.56, 1.60] to 2.15 [95% CI 2.09, 2.20]), Anti-Ps Penicillin (0.19 [95% CI 0.18,0.20] to 0.26 [95% CI 0.25, 0.27]), NS Different Cephalosporin (0.44 [95% CI 0.44, 0.45] to 0.81 [95% CI 0.80, 0.82]), NS Similar Cephalosporin (0.53 [95% CI 0.49, 0.57] to 0.84 [95% CI 0.79, 0.89]), and NS Penicillin (0.15 [95% CI 0.14, 0.16] to 0.32 [95% CI 0.31, 0.34]) (Table 3, Figure S3-S5). The PR did not change significantly for aminoglycosides (1.40 [95% CI 1.33, 1.47] to 1.31 [95% CI 1.20, 1.43]) nor clindamycin (4.01 [95% CI 3.91, 4.12] to 3.91 [95% CI 3.76, 4.07]) (Table 3, Figure S3).

## Discussion

In this analysis of a national sample of acute care admissions, the prevalence of PCN-class allergies reported at the time of acute care admission decreased from 12.99% in 2011-2012 to 11.20% in 2021-2022. In general, non-βL antibiotic use decreased and narrow-spectrum βL agent use increased, regardless of PCN-class allergy status. In regard to broad-spectrum βLs, use decreased for Anti-Ps Penicillin, Carbapenem, and Monobactam agents; and increased for Anti-Ps Cephalosporin agents. Prescribing differences in “PCN-class allergy only” and “No antibiotic allergy” admissions, as measured by the PR for antibiotic use, decreased for Anti-MRSA and βL agents, remained stable for aminoglycosides and clindamycin, and increased for fluoroquinolones.

Our study updates the prevalence of PCN-class allergies reported at the time of admission to acute care VHA facilities, which was previously estimated to be 13% based on an analysis of 10,858,398 admissions between 2000 and 2014.^1^ Despite differences in inclusion criteria, the prevalence was similar during the years in which our study periods overlapped (2010-2014). The decline in reported PCN-allergy prevalence over time is consistent with previous time-trend analyses of VHA and other regional healthcare system data.^1,33^ The reason for this decline has not been extensively investigated, partly because reports regarding national trends of PCN-allergy prevalence are limited. Because older age is associated with higher prevalence of βL allergy,^34^ one possibility is that this decline is an artifact of higher mortality as patients age. However, for this to be true, βL allergy needs to be associated with higher mortality, and this has not been consistently shown.^35^ Notably, most interventions aimed at clarifying or discontinuing PCN allergy labels at VHA facilities, including the National Allergy to Beta-Lactam Evaluation (ABLE) program,^19^ were not implemented until after 2014.^7,20–28^ Our study did not compare reported PCN-allergy prevalence at sites with different PCN allergy evaluation strategies and cannot directly measure the impact of these strategies. However, by analyzing more recent data, our findings offer insight into the trajectory of this trend at the national level.

Data regarding the impact of the PCN allergy label on inpatient antibiotic use at VHA facilities is limited to small single-center studies. In a study of 403 Veterans hospitalized between 2006 and 2015, PCN-allergic patients were more likely to receive carbapenems (5.3 vs 0.3%) and fluoroquinolones (61.4 vs 26.3%).^7^ In another study of patients admitted to the Memphis VA, 95 inpatients with a reported βL (96% PCN) allergy received non-preferred antibiotic regimens, which consisted primarily of fluoroquinolones and vancomycin, 48% of the time.^28^ Studies of PCN-allergic Veterans treated in outpatient and perioperative settings have found that they were more likely to receive clindamycin, fluoroquinolones, and vancomycin.^18,36^ Our study found similar associations across the VHA with Aminoglycoside, Anti-MRSA, Clindamycin, Fluoroquinolone, Anti-Ps Cephalosporin, Carbapenem, and Monobactam agent use being more likely in “PCN-class allergy only” admissions. Additionally, we found that inpatients that reported a PCN-class allergy were less likely to receive cefazolin and non-pseudomonal third generation cephalosporins, which can be safely used even in patients with severe IgE-mediated hypersensitivity reactions to PCN.^17^ Unsurprisingly, penicillins and first- and second-generation cephalosporins were less likely to be administered in “PCN-class allergy only” admissions.

Our study showed some encouraging trends. First, we observed a steady decline in non-βL antibiotic use and a steady rise in narrow-spectrum βL use (primarily for NS Different Cephalosporin agents), regardless of PCN-class allergy status. The trend in broad-spectrum βL use was more varied; Anti-Ps Penicillin, Carbapenem, and Monobactam agent use decreased modestly, and Anti-Ps Cephalosporin use increased over time. These findings confirm previous reports of decreasing inpatient broad-spectrum antibiotic use following the establishment of the national VHA Antimicrobial Stewardship initiative in 2008.^37,38^ Second, use of cefazolin and non-pseudomonal third-generation cephalosporins in “PCN-class allergy only” admissions increased from 9.01 to 20.47% during the study period, which is consistent with reports of increased alternate βL use among PCN-allergic patients after implementation of PCN allergy evaluation programs at individual VHA medical centers.^3,24^ Use of penicillins and earlier generation cephalosporins also increased, albeit more modestly. Reports of narrow-spectrum βL use in VHA facilities are limited; a 5-year analysis of 134 CLCs noted a relatively stable trend in first- and second-generation cephalosporin use.^39^ Third, the decreasing difference in antibiotic use patterns between “PCN-class allergy only” and “No antibiotic allergy” admissions raises the possibility that progress is being made in mitigating the effect of the PCN allergy label. However, this cannot be ascertained without additional analyses of specific interventions and clinical outcomes. Despite an overall decline in non-βL agent use, patients that reported a PCN-class allergy on admission in 2021-2022 were as likely as they were in 2011-2012 to receive an aminoglycoside or clindamycin, and more likely to receive a fluoroquinolone. The reason behind these trends is unclear but they represent potential opportunities for additional mitigation of the impact of the PCN allergy label during “PCN-allergy only” hospitalizations.

This study has several strengths. By using a recent sample of millions of acute care admissions from more than 120 locations across the country, we are providing the most up-to-date estimate of the national prevalence of PCN and other βL allergies among hospitalized Veterans and the first multi-site analysis of inpatient antibiotic use at VHA acute care facilities by PCN-class allergy status. The reported trends can serve to identify areas of focus for future analyses or interventions related to the impact of the PCN allergy label on antibiotic selection. Additionally, the use of nationally standardized allergy and antibiotic administration records increases the accuracy and generalizability of our findings. Lastly, although our findings are specific to VHA facilities, Veterans regularly obtain care in the community and the impact of their PCN allergy labels may carry over to those institutions.

In addition to the limitations inherent to a cross-sectional study design, our study had limitations specific to how allergy and antibiotic administration records are entered into our EHR. First, although Veterans are also admitted to community hospitals^29^ and data from those institutions may grant additional allergy and antibiotic use data, we limited our analysis to data from VHA facilities because of the added complexity involved in integrating community care data into our database. Additionally, since VHA antibiotic stewardship policies have no direct effect on community hospitals, analyzing data from those facilities was outside of this project’s scope. Second, the inability of VHA stations to automatically update allergy records across all stations means that an allergy entered in one station does not appear in a different station until it is re-entered at that station. Because of this, it is possible to miscount allergies for patients that register at multiple stations. The impact of this issue on inpatients should be limited, however, as allergy records are generally updated prior to any Emergency Department or inpatient encounter. To ensure the accuracy of this assumption, the authors (FL, HH, EH, and LTP) reviewed the entire allergy and admission history (across all VHA stations) of a random sample of patients and found no inconsistencies in how active allergies were captured by our database query. Additionally, by counting antibiotic allergies only if they were present in the admitting station’s EHR at the time of admission, we simulated the information available to the admitting prescribers at the time of antibiotic selection. Lastly, in VHA facilities, BCMA is not always used in hemodialysis and procedural locations.^40^ The authors reviewed a random sample of 60 hospitalizations and found that in 1 (1.67%), our query did not capture a dose of vancomycin that was administered in a dialysis unit.

In conclusion, the prevalence of reported PCN-class allergies is decreasing and the use of narrower-spectrum βLs is increasing among hospitalized Veterans. Additionally, differences in antibiotic administration between hospitalizations with and without a reported PCN-class allergy are decreasing. However, Veterans who report a PCN-class allergy on admission are still more likely to receive aminoglycosides, clindamycin, and fluoroquinolones. Our findings can serve to identify areas of focus for more specific analyses of the impact of PCN allergy evaluation programs and inform the implementation of those programs.

## Supporting information

Liu et al. supplementary material Liu et al. supplementary material

## Supplementary material

For supplementary material accompanying this paper visit http://doi.org/10.1017/ash.2025.11. click here to view supplementary material
