# Supplementary material for: Patterns of antimicrobial use among hospitalized Veterans with and without a penicillin-class allergy
Source: Antimicrob Steward Healthc Epidemiol. 2025 Feb 25;5(1):e57. doi: 10.1017/ash.2025.11 (PMC11869061; doi:10.1017/ash.2025.11)
Supplement: Liu et al. supplementary material [file S2732494X25000117sup001.docx]

**Supplementary material**

**Supplementary Table S1. Inclusion and Exclusion Criteria.**

| **Inclusion criteria** | 1. Adult patients. 2. Hospitalized to an acute care service (Cardiology, Medicine, Neurology, Oncology, Rehabilitation Medicine, Spinal Cord Injury, Surgery) between 01/01/11 and 12/31/22. |
| --- | --- |
| **Exclusion criteria** | 1. Admitted to a facility that transitioned to the Cerner Oracle electronic health record (EHR) during the study period (Central Ohio VA (Columbus, OH), Jonathan Wainwright VA (Walla Walla, WA), Roseburg VA (Roseburg, OR), Spokane VA (Spokane, WA)). 2. Admitted to any of the following services: Blind Rehabilitation, Community Living Center, Respite Care, Psychiatry, Domiciliary. 3. Incomplete hospitalizations (i.e., patient admitted but not discharged by the end of the study period) |

**Supplementary Table S2. General definitions.**

| **Acute care admission type** | - Observation only: Patient admitted to observation medical specialty without a subsequent full admission. - Full only: Patient admitted to non-observation medical specialty without a preceding observation admission. - Combination: Patient admitted to sequentially to observation and non-observation medical specialties. The discharge date of the observation admission equals the admission date of the non-observation admission. Duration of stay and antibiotics is calculated from the admission date of the observation admission to the discharge date of the non-observation admission. |
| --- | --- |
| **Active antibiotic allergy** | - Allergy Origination Date/Time at Admitting station before Admission Date/Time at Admitting station.   AND  Allergy Entered in Error Date/Time at Admitting station is NULL or after Admission Date/Time at Admitting station. |

**Supplementary Table S3. Antibiotic allergy group definitions.**

| **Antibiotic allergy group** | **Allergy database search terms** |
| --- | --- |
| **Penicillin** | - Penicillin, Bicillin, PCN; alternate/erroneous spellings: Pencillin, Pencilllin, Penecillin, Penicllin, Pennicillin, PEN VK, PENVK, PNC, PEN |
| **Amoxicillin/ Ampicillin** | - Amoxicillin, Augmentin - Ampicillin, Polycillin, Unasyn - Penicillin Amino Derivatives |
| **Other Synthetic Penicillin** | - Dicloxacillin, Diclocil, Dicloxicil - Nafcillin, Nafcil, Nallpen - Oxacillin, Bactocil - Carbenicillin - Piperacillin, Zosyn - Ticarcillin - Penicillinase-resistant penicillins |
| **Cefazolin** | - Cefazolin, Ancef, Kefzol |
| **Cephalosporin, 1st/2nd generation** | - Cefaclor, Ceclor, Raniclor - Cefadroxil, Duricef - Cefalotin, Keflin - Cefonicid, Monocid, Sintocef - Cefotetan, Apatef, Cefotan - Cefoxitin, Mefoxin - Cefprozil, Cefzil - Cefuroxime, Ceftin, Zinacef - Cephalexin, Keflex - Cephalosporin 2nd Generation |
| **Cephalosporin, 3rd generation** | - Cefdinir, Omnicef - Cefixime, Suprax - Cefotaxime, Claforan - Cefpodoxime, Vantin - Ceftazidime, Fortaz, Tazicef - Ceftriaxone, Ceftrisol, Rocephin - Cephalosporin 3rd generation |
| **Cephalosporin, 4th generation** | - Cefepime, Maxipime - Cefpirome, Broact, Cefir, Cefrom, Keiten |
| **Cephalosporin, other** | - Cefiderocol, Fejtroja - Ceftaroline, Teflaro - Ceftolozane-tazobactam, Zerbaxa - Ceftazidime-avibactam, Avycaz |
| **Aztreonam** | - Aztreonam, Azactam |
| **Carbapenems** | - Doripenem, Doribax - Ertapenem, Invanz - Imipenem, Primaxin, Recabrio - Meropenem, Merrem, Vabomere |
| **Aminoglycoside** | - Amikacin - Gentamicin; alternate/erroneous spellings: Gentamycin - Streptomycin - Tobramycin - Aminoglycoside |
| **Clindamycin** | - Clindamycin, Cleocin |
| **Daptomycin** | - Daptomycin, Cubicin |
| **Fluoroquinolone** | - Ciprofloxacin, Cetraxal, Ciloxan, Ciproxin, Alternate and erroneous spellings: Cipro, Cipro I.V. - Delafloxacin, Baxdela - Gatifloxacin, Tequin - Gemifloxacin, Factive - Levofloxacin, Levaquin; alternate/erroneous spellings: Levoquin - Moxifloxacin, Avelox - Quinolone |
| **Macrolide** | - Azithromycin, Z-pak, Zithromax; alternate/erroneous spellings: Zithomax - Clarithromycin - Erythromycin |
| **Metronidazole** | - Metronidazole, Flagyl |
| **Oxazolidinone** | - Linezolid, Zyvox - Tedizolid, Silvextro |
| **Tetracycline** | - Doxycycline, Vibramycin - Eravacycline, Xerava - Minocycline, Dynacin, Minocin, Ximino - Omadacycline, Nuzyra - Tetracycline - Tigecycline, Tygacil |
| **TMP-SMX** | - Co-triamoxazole, sulfamethoxazole, trimethoprim, Bactrim, Septra, TMP, SMX |
| **Vancomycin** | - Vancomycin (IV only), Firvanq |
| **Other specified antibiotic** | - Colistin, polymyxin - Dalbavancin, Dalvance - Fidaxomicin, Dificid - Nitrofurantoin, Macrobid, Macrodantin, Microdantin - Oritavancin, Orbactiv - Quinipristin-dalfopristin - Rifabutin, Rifampin, Rifapentine - Rifaximin - Telavancin, Vibativ - Telithromycin |
| **Unspecified antibiotic** | - Antibiotic - Antimicrobial |
| **Unspecified beta-lactam** | - Beta-lactam - Penicillins and beta-lactams |
| **Unspecified cephalosporin** | - Cephalosporin |
| **Unspecified sulfa** | - Sulfa drug, sulfisoxazole, sulfonamide; alternate/erroneous spellings: Sulfa + Free Text, sulfa, sulfa, sulpha |

**Supplementary Table S4. Administered antibiotic group definitions.**

| **Administered antibiotic group definitions** | **Bar-code administration (BCMA) record database search terms** |
| --- | --- |
| **Aminoglycosides**^1^ | - Amikacin - Gentamicin - Streptomycin - Tobramycin |
| **Anti-MRSA agents** | - Dalbavancin, Dalvance - Daptomycin, Cubicin - Linezolid, Zyvox - Oritavancin, Orbactiv - Quinupristin, Dalfopristin, Synercid - Tedizolid, Silvextro - Telavancin, Vibativ - Vancomycin^3^, Firvanq |
| **Anti-pseudomonal cephalosporins** | - Cefepime, Maxipime - Ceftazidime, Fortaz, Tazicef |
| **Anti-pseudomonal penicillins** | - Piperacillin-tazobactam, Zosyn |
| **Carbapenems**^2^ | - Doripenem, Doribax - Ertapenem, Invanz - Imipenem, Primaxin - Meropenem, Merrem |
| **Clindamycin**^1^ | - Clindamycin, Cleocin |
| **Fluoroquinolones**^1^ | - Ciprofloxacin, Cetraxal, Ciloxan, Ciproxin - Delafloxacin, Baxdela - Gatifloxacin, Tequin - Gemifloxacin, Factive - Levofloxacin, Levaquin - Moxifloxacin, Avelox |
| **Monobactams** | - Aztreonam, Azactam |
| **Narrow-spectrum structurally different cephalosporins** | - Cefazolin, Ancef, Kefzol - Cefotetan, Apatef, Cefotan - Cefoxitin, Mefoxin - Cefuroxime, Ceftin, Zinacef - Cefdinir, Omnicef - Cefixime, Suprax - Cefotaxime, Claforan - Cefpodoxime, Vantin - Ceftriaxone, Ceftrisol, Rocephin |
| **Narrow-spectrum structurally similar cephalosporins** | - Cefaclor, Ceclor, Raniclor - Cefadroxil, Duricef - Cefprozil, Cefzil - Cephalexin, Keflex |
| **Narrow-spectrum penicillins** | - Amoxicillin - Amoxicillin-clavulanic acid, Amoxicillin-clavulanate, Augmentin - Ampicillin, Polycillin - Ampicillin-sulbactam, Unasyn - Dicloxacillin, Diclocil, Dicloxicil - Nafcillin, Nafcil, Nallpen - Oxacillin, Bactocil - Penicillin G, Penicillin V, Bicillin |

^1^ Excluded ophthalmic, otic, and topical formulations;  ^2^ Excluded combination of carbapenem and β-lactamase inhibitor; ^3^ Excluded oral and rectal formulations.

**Supplementary Table S5. Percent of acute care admissions between 2011 and 2022 where patients reported a non-β-lactam allergy as their only antibiotic allergy or as one of multiple antibiotic allergies.**

|  | **All Admissions**^1^  **N=6,541,299** |
| --- | --- |
| **Single allergy reported at admission** |  |
| Aminoglycoside | 3,527 (0.05%) |
| Clindamycin | 12,443 (0.19%) |
| Daptomycin | 1,233 (0.02%) |
| Fluoroquinolone | 70,523 (1.07%) |
| Macrolide | 30,605 (0.47%) |
| Metronidazole | 5,540 (0.08%) |
| Oxazolidinone | 1,559 (0.02%) |
| Tetracycline | 34,861 (0.53%) |
| TMP-SMX | 64,645 (0.99%) |
| Unspecified Sulfa | 140,209 (2.14%) |
| Vancomycin | 17,286 (0.26%) |
| Other Specified Antibiotic Allergy | 5,806 (0.09%) |
| Unspecified Antibiotic | 12 (<0.01%) |
| **Multiple allergies reported at admission**^1^ |  |
| Aminoglycoside | 5,648 (0.09%) |
| Clindamycin | 23,236 (0.36%) |
| Daptomycin | 1,520 (0.02%) |
| Fluoroquinolone | 72,356 (1.11%) |
| Macrolide | 39,429 (0.60%) |
| Metronidazole | 8,846 (0.14%) |
| Oxazolidinone | 2,455 (0.04%) |
| Tetracycline | 39,482 (0.60%) |
| TMP-SMX | 60,563 (0.93%) |
| Unspecified Sulfa | 99,915 (1.53%) |
| Vancomycin | 21,879 (0.33%) |
| Other Specified Antibiotic Allergy | 10,347 (0.16%) |
| Unspecified antibiotic | 67 (<0.01%) |

Abbreviations: LOS Length of stay, SD Standard deviation

^1^ Includes patients that reported multiple antibiotic allergies at the time of admission; could report a single or multiple non-β-lactam allergies as part of those multiple antibiotic allergies.

**Supplementary Figure S1. Percent of acute care admissions where a penicillin-class allergy was reported either as the only antibiotic allergy or as one of multiple antibiotic allergies by age group (≥65 and <65 years).**

**
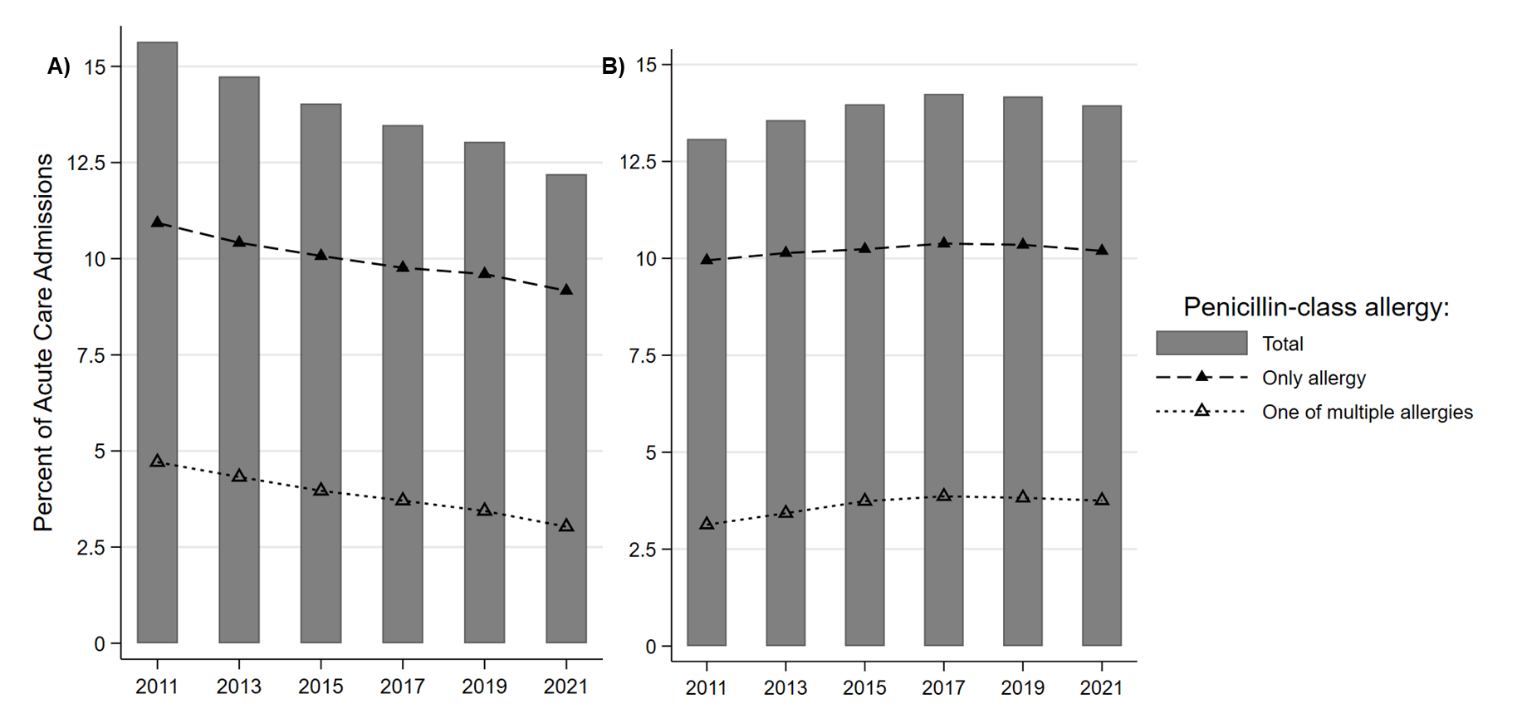
** Percent of acute care admissions where a penicillin-class allergy was reported either as the only antibiotic allergy or as one of multiple antibiotic allergies for age at admission ≥65 (A) and <65 (B).
***** Each bar represents a 2-year period. The x-axis shows the first year of that 2-year period (e.g., 2011 includes 2011 and 2012).

**Supplementary Figure S2. Percent of acute care admissions where a penicillin-class allergy was reported either as the only antibiotic allergy or as one of multiple antibiotic allergies by region.**


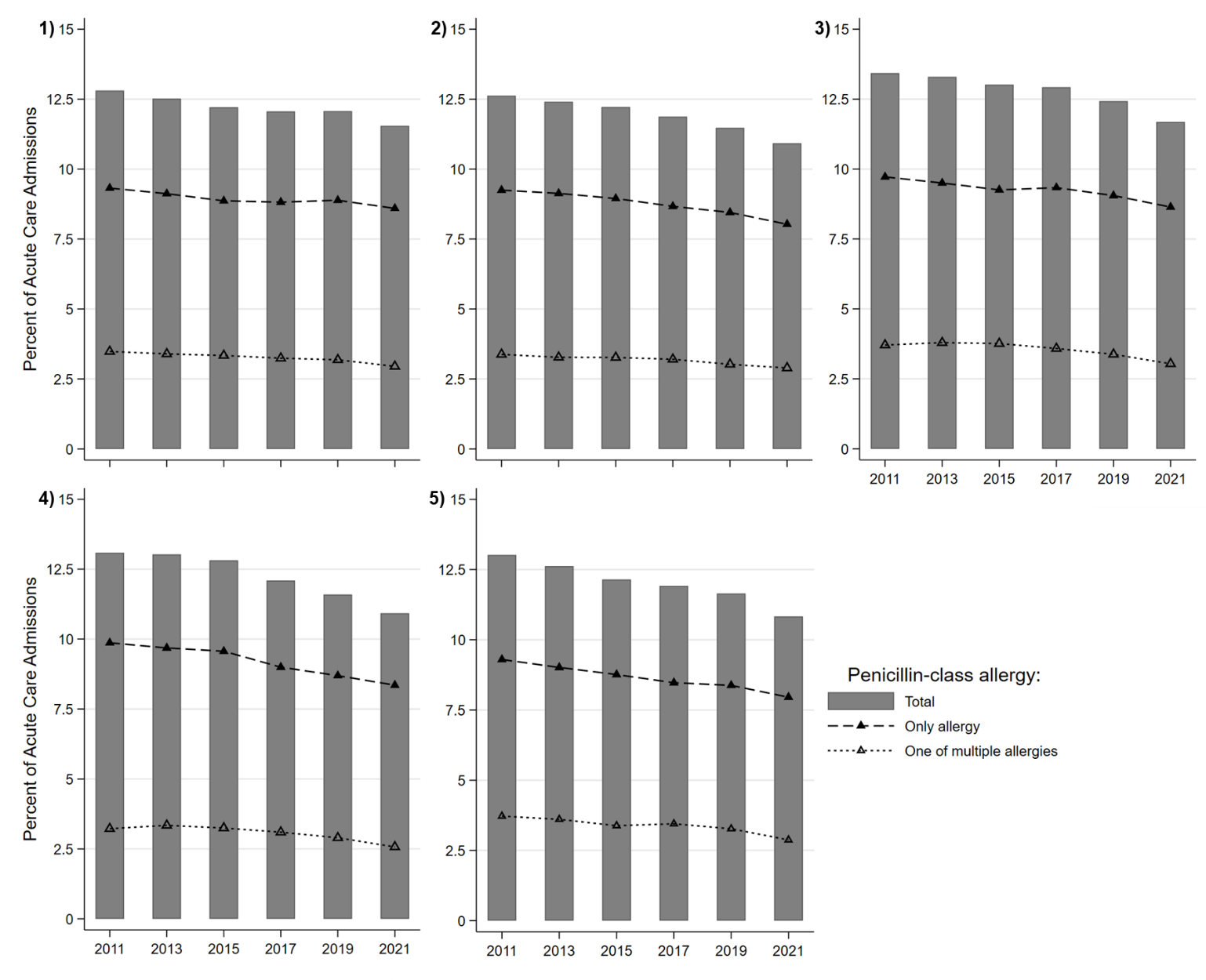


Percent of acute care admissions where a penicillin-class allergy was reported either as the only antibiotic allergy or as one of multiple antibiotic allergies for regions 1-5. Region 1: CT, DC, DE, MA, MD, ME, NC, NH, NJ, NY, PA, RI, VA, VT, WV; Region 2: AL, FL, GA, KY, PR, SC TN; Region 3: IA, IL, IN, KS, KY, MI, MN, MO, ND, NE, OH, SD, WI; Region 4: AR, CO, LA, MS, MT, OK, TX, UT, WY; Region 5: AZ, CA, HI, ID, NM, NV, OR, WA.
***** Each bar represents a 2-year period. The x-axis shows the first year of that 2-year period (e.g., 2011 includes 2011 and 2012).

**Supplementary Figure S3. Non-β-lactam antibiotic use over time in acute care admissions with and without a reported penicillin-class allergy.**


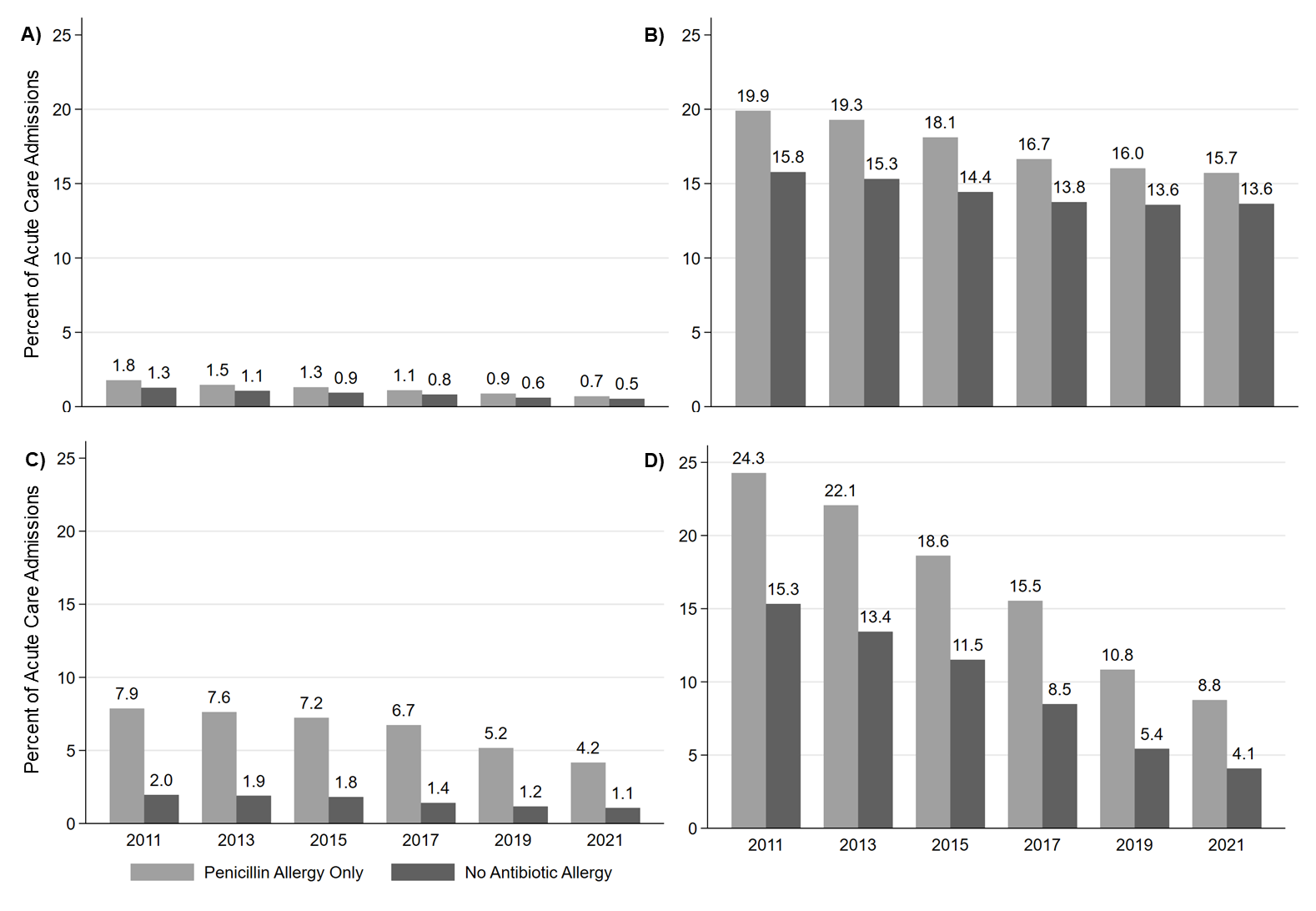


Percent of acute care admissions with and without a reported penicillin-class allergy in which patients received ≥1 dose of an Aminoglycoside (A), Anti-MRSA (B), Clindamycin (C), and Fluoroquinolone (D) agent.
* Each bar represents a 2-year period. The x-axis shows the first year of that 2-year period (e.g., 2011 includes 2011 and 2012).

**Supplementary Figure S4. Broad-spectrum β-lactam antibiotic use over time in acute care admissions with and without a reported penicillin-class allergy.**

**
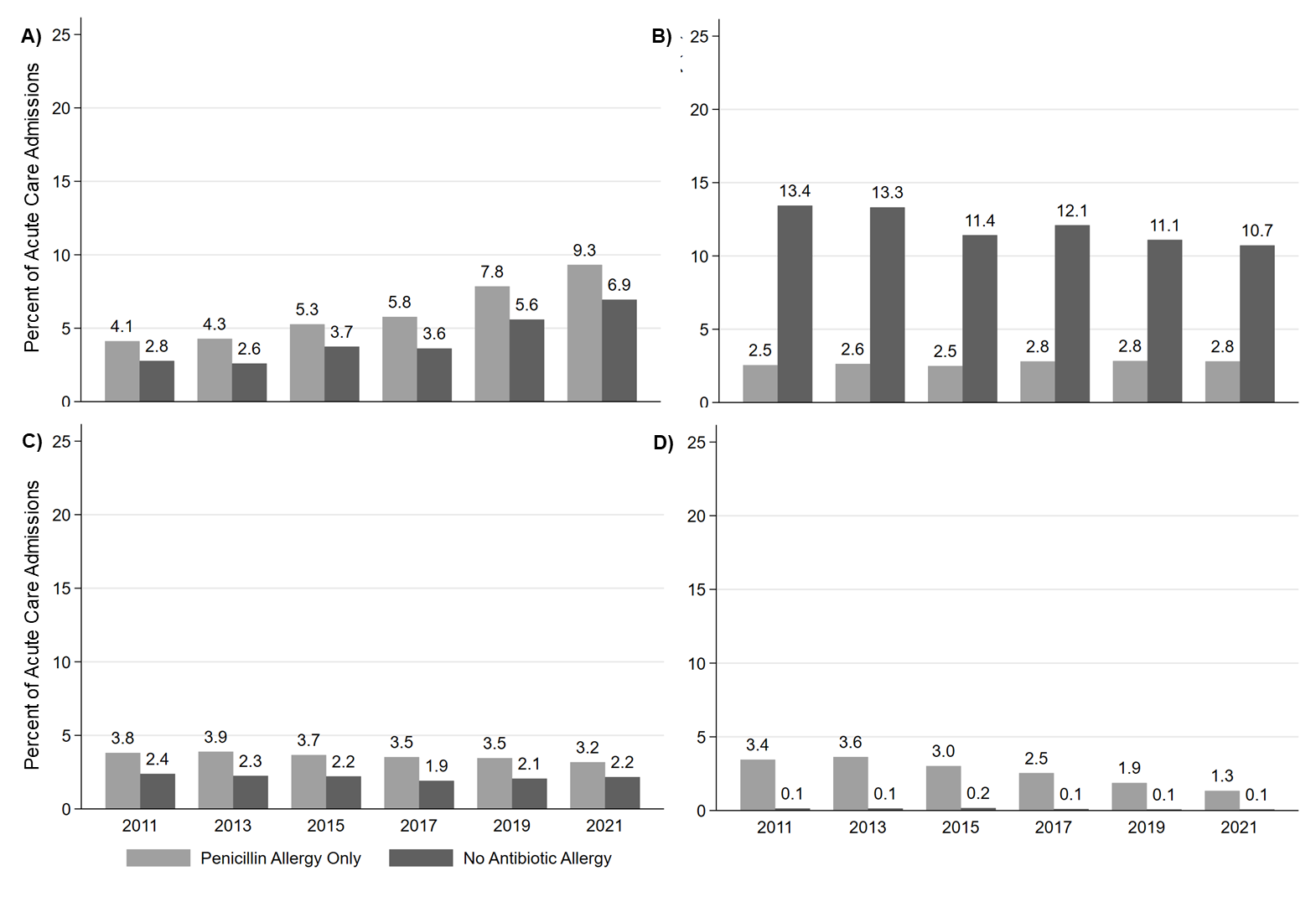
**

Percent of acute care admissions with and without a reported penicillin-class allergy in which patients received ≥1 dose of an Anti-Pseudomonal Cephalosporin (A), Anti-Pseudomonal Penicillin (B), Carbapenem (C), and Monobactam (D) agent.
* Each bar represents a 2-year period. The x-axis shows the first year of that 2-year period (e.g., 2011 includes 2011 and 2012).

**Supplementary Figure S5. Narrow-spectrum β-lactam antibiotic use over time in acute care admissions with and without a reported penicillin-class allergy.**


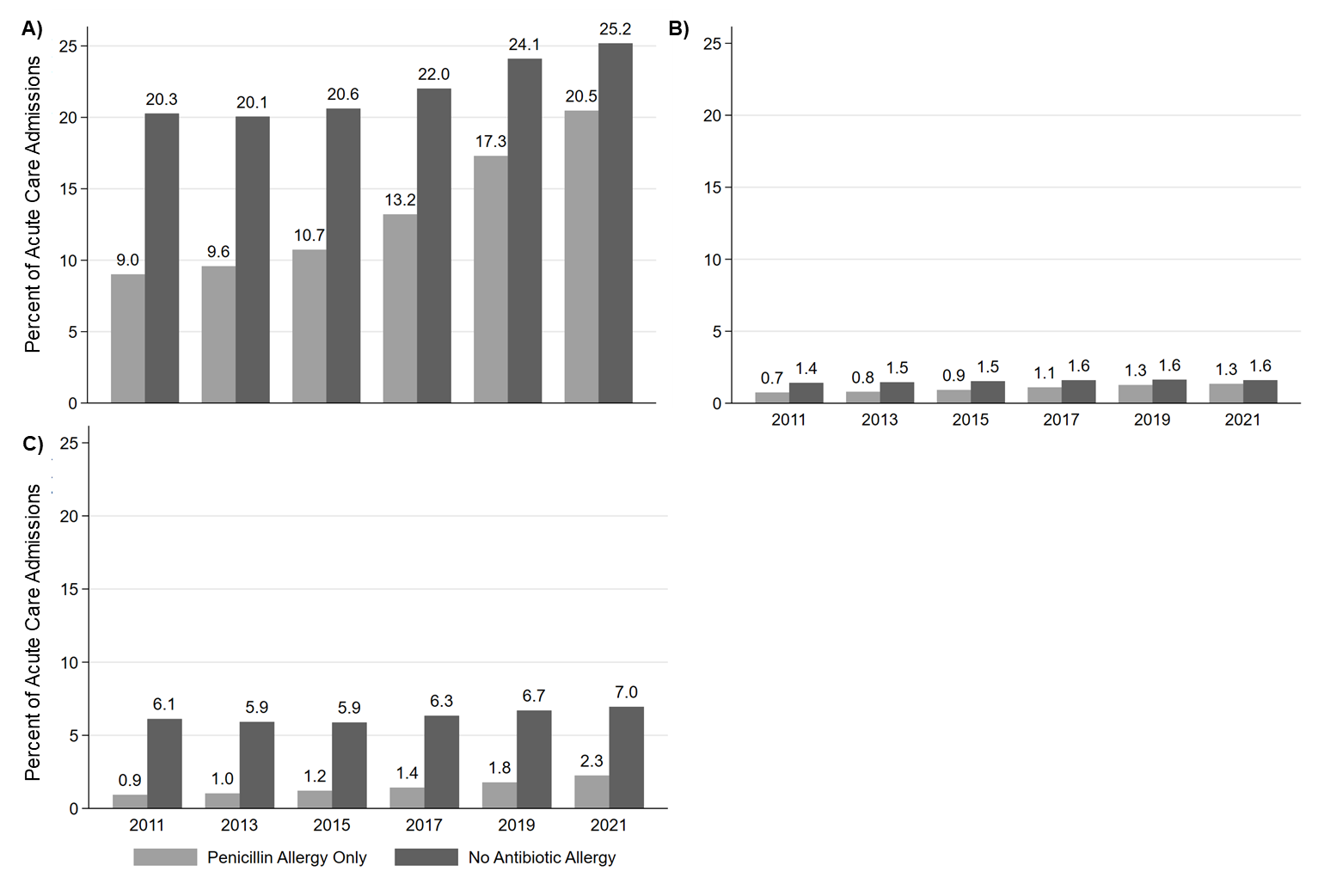


Percent of acute care admissions with and without a reported penicillin-class allergy in which patients received ≥1 dose of a Narrow-Spectrum Structurally Different Cephalosporin (A), Narrow-Spectrum Structurally Similar Cephalosporin (B), and Narrow-Spectrum Penicillin (C) agent.
* Each bar represents a 2-year period. The x-axis shows the first year of that 2-year period (e.g., 2011 includes 2011 and 2012).
